# Haplotype-Based Analysis of *KIR*-Gene Profiles in a South European Population—Distribution of Standard and Variant Haplotypes, and Identification of Novel Recombinant Structures

**DOI:** 10.3389/fimmu.2020.00440

**Published:** 2020-03-17

**Authors:** Elisa Cisneros, Manuela Moraru, Natalia Gómez-Lozano, Aura Muntasell, Miguel López-Botet, Carlos Vilches

**Affiliations:** ^1^Immunogenetics and Histocompatibility, Instituto de Investigación Sanitaria Puerta de Hierro Segovia de Arana, Madrid, Spain; ^2^Hospital del Mar Medical Research Institute (IMIM), Barcelona, Spain; ^3^Department of Experimental and Health Sciences, University Pompeu Fabra, Barcelona, Spain

**Keywords:** copy-number variation, genes, haplotypes, KIR, NK cells, polymorphism

## Abstract

Inhibitory Killer-cell Immunoglobulin-like Receptors (KIR) specific for HLA class I molecules enable human natural killer cells to monitor altered antigen presentation in pathogen-infected and tumor cells. *KIR* genes display extensive copy-number variation and allelic polymorphism. They organize in a series of variable arrangements, designated *KIR* haplotypes, which derive from duplications of ancestral genes and sequence diversification through point mutation and unequal crossing-over events. Genomic studies have established the organization of multiple *KIR* haplotypes—many of them are fixed in most human populations, whereas variants of those have less certain distributions. Whilst *KIR*-gene diversity of many populations and ethnicities has been explored superficially (frequencies of individual genes and presence/absence profiles), less abundant are in-depth analyses of how such diversity emerges from *KIR*-haplotype structures. We characterize here the genetic diversity of KIR in a sample of 414 Spanish individuals. Using a parsimonious approach, we manage to explain all 38 observed *KIR*-gene profiles by homo- or heterozygous combinations of six fixed centromeric and telomeric motifs; of six variant gene arrangements characterized previously by us and others; and of two novel haplotypes never detected before in Caucasoids. Associated to the latter haplotypes, we also identified the novel transcribed KIR2DL5B^*^0020202 allele, and a chimeric *KIR2DS2*/*KIR2DL3* gene (designated KIR2DL3^*^033) that challenges current criteria for classification and nomenclature of *KIR* genes and haplotypes.

## Introduction

Human killer-cell immunoglobulin-like receptors (KIR) are a diverse and polymorphic family of glycoproteins that convey inhibitory or activating signals to subpopulations of NK and T lymphocytes upon recognition of their ligands, mainly HLA class I allotypes (1). In coordination with multiple other activating and inhibitory receptors for HLA class I and non-HLA molecules, KIR regulate the function of cytotoxic lymphocytes, providing them with a capacity to sense modifications of HLA expression on potential target cells (2, 3).

**Figure 1:**
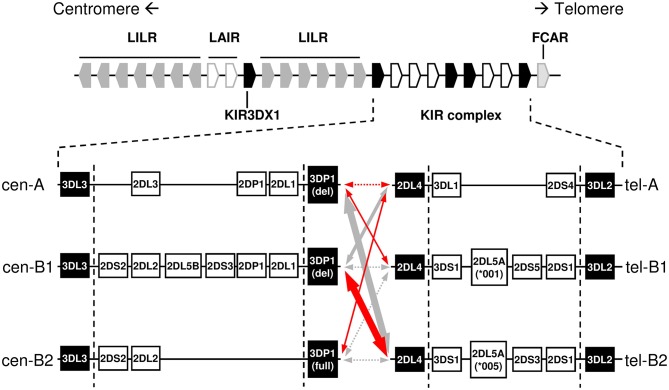
Centromeric and telomeric *KIR-*gene haplotypes commonly observed in Caucasoids, and their linkage disequilibrium in Spanish individuals. Positive and negative relative linkage disequilibrium values are represented with red and gray arrows, respectively. Dotted lines indicate non-statistically significant LD, whilst thickness of solid lines indicates the level of statistical significance (*p* < 0.05/0.01/0.0001). Conserved “framework” genes are represented as solid boxes. Genes and intergenic spaces are not depicted to scale.

KIR repertoires expressed by NK cells of different individuals display a conspicuous phenotypic and functional diversity, genetically determined in its greatest part. KIR are encoded in a ~100–250 Kbp complex on chromosome 19q13.4, where variable combinations of 15 *KIR* genes and 2 pseudogenes arrange in a head-to-tail orientation, separated by intergenic regions of only ~2.5 Kbp (4). Another ancestral gene, *KIR3DX1* (5), lays ~178 Kbp upstream of the *KIR* complex, in the middle of the *LILR*-*LAIR* gene cluster (Figure 1).

The *KIR* complex is extremely diverse due to allelic polymorphism and gene copy-number variation (CNV). Only three “framework” regions of the *KIR* complex are relatively well-conserved in their gene content: the genes at the 5′ and 3′ ends (*KIR 3DL3* and *3DL2*, respectively), and a central cluster formed by *KIR 3DP1* and *2DL4*. These framework *KIR* genes define the limits of two intervals, centromeric (5′) and telomeric (3′), containing variable combinations of the other genes (4, 6–10). Certain gene arrangements or “motifs” are particularly common within each of those intervals (Figure 1); in turn, the different centromeric and telomeric gene motifs are seen in any combinatorial association, possibly owing to a recombination hot-spot between *KIR 3DP1* and *2DL4*.

Restriction-fragment length polymorphism studies published in 1997 sorted *KIR* genotypes into categories “A” and “B,” based on variable presence of a 24 Kbp-long *Hind*III band, later shown to derive from the *KIR2DL5* gene (11, 12). This definition was then refined and adapted (13), so that “A haplotype” now officially designates a nearly fixed combination of seven genes and pseudogenes, encoding the HLA-C-specific KIR 2DL3 and 2DL1 in the variable centromeric interval, and 3DL1 and 2DS4 in the telomeric one. In contrast, “B” designates collectively a vast array of haplotypes bearing any additional *KIR* gene (even when they also have, as it often happens, parts of an A haplotype). Of immunologic relevance, the “A” haplotype encodes inhibitory KIR for all known HLA ligands; and, at most, a single activating KIR expressed on the NK-cell surface, KIR2DS4 being often represented by an aberrant allele (14). In contrast, “B” haplotypes are distinguished by one or more of the following features: they encode several activating KIR; lack one or more genes for the aforementioned inhibitory KIR; and/or carry *KIR2DL5*, of uncertain biological role (15). This diversity appears to influence many human health conditions (16).

Certain neighbor *KIR* genes tend to appear strongly linked in the same haplotype. Noteworthy among those is the pair formed by *KIR 2DL5* and either *2DS3* or *2DS5*, these being inherited like allotypes of the same locus (17). This *KIR 2DL5*–*2DS3*/*S5* cluster duplicated and diversified jointly during human evolution, and is seen on either or both of the centromeric or the telomeric intervals of many B haplotypes (8, 15, 17, 18). Existence of two such long and highly similar stretches of sequence favored further asymmetric recombination between the paralogous regions. This resulted in expanded and shortened haplotypes bearing tandem duplications or deletions of the intervening genes, as shown by us and others (9, 10, 15, 19–24). Additional consequences of asymmetric recombination are novel fusion genes encoding chimeric KIR that blend structural and functional features of their parent receptors.

Initial studies of human *KIR* genotypes faced this vast polymorphism without previous knowledge of the structure and forms of variation of the *KIR*-gene complex, revealing multiple *KIR*-gene and allele profiles in different individuals, which could give a false impression of randomness (11, 25). Order, in the form of knowledge on common and variant patterns of association between *KIR* genes and alleles, emerged from subsequent studies of population groups and families; and from phasing and physical mapping of partial and complete *KIR*-gene haplotypes by DNA sequencing (4, 6–10, 26–29). Those studies were complemented by others focused on estimation of CNV and allelic diversity (23, 24, 30–39). In parallel, *KIR*-gene profiles were studied in many healthy and diseased populations and ethnic groups worldwide (40). However, many those population studies have benefited surprisingly little from knowledge gained in the last years on the structure and patterns of variability of the *KIR* gene complex, and, for many human populations, only collections of *KIR*-gene profiles and superficial analyses of the basic variations are available. Here, we have applied current knowledge of *KIR*-gene arrangements to a comprehensive analysis of the gene profiles observed in a European Mediterranean population.

## Materials and Methods

### Samples

Genomic DNA was isolated using standard methods from peripheral blood or mononuclear cell (PBMC) suspensions, obtained by Ficoll-Hypaque density gradient centrifugation (Lymphoprep, Axis-Shield PoC AS, Oslo, Norway), from 414 unrelated voluntary donors recruited in our centers in Madrid and Barcelona, mostly of Caucasoid origin; only known exceptions were two donors of mixed Hispanic/Amerindian ancestry, neither of whom contributed novel or variant gene profiles. Complementary DNA was synthesized with the AffinityScript Multiple Temperature cDNA Synthesis Kit (Agilent Technologies, Santa Clara, CA, USA) from 400 ng of total RNA, extracted from PBMCs of selected donors using the RNeasy Plus Mini kit (Qiagen GmbH, D-40724, Hilden, Germany).

### *KIR* Genotyping

*KIR* genes, structural variants of *KIR 2DS4, 2DL5*, and *3DP1*, and the hybrid alleles 2DS2^*^005 and 3DP1^*^004 were typed by PCR with sequence-specific primers (SSP), as previously described (22, 41–43). Non-standard *KIR*-gene profiles were confirmed utilizing a commercial reverse oligonucleotide probe-hybridization method based on the Luminex xMap Technology (LabType SSO Test, One Lambda Inc., Canoga Park, CA). To verify presence of an expanded haplotype in a 3DP1^*^004^−*ve*^ donor, existence of three *KIR3DL1/S1* alleles was verified by sequence-based typing of exons 3–5 in two overlapping amplicons. Each of these was generated with Advantage-2 polymerase (BD-Clontech, Palo Alto, CA, USA) and primer mixes F153/Rt624 (5′–tggtcaggacaarccctt−3′, exon 3; 5′–aggtccctgcaagggcaa−3′, exon 4) or Fg539/Rc959 (5′–acttctttctgcacaaagagg−3′, exon 4; 5′–cmactcgtagggagagtg−3′, exon 5). PCR conditions were: 1 min at 95°C, then 10 cycles of 30 s at 94°C, 30 s at 64°C and 120 s at 72°C; 20 cycles of 30 s at 94°C, 30 s at 60°C and 120 s at 72°C; final elongation of 10 min at 72°C. Exon sequences were determined using internal primers (not shown). To assess presence of a *KIR3DL1/L2* chimera (3DL1^*^060) (44, 45), its third through fifth and seventh through ninth exons were amplified separately using, respectively, primer mixes Fi2c−201/Ri5+305 (5′–tctagtaagagttgcttctc−3′, intron 2; 5′–atgggcttctgggaaatgga−3′, intron 5); and Fi6g−235/Ra1461 (5′–gagaaagcaggagaaagctg−3′, intron 6; 5′–gttcattggatctggcaacct−3′, exon 9). PCR conditions were: for exons 2–5, 2 min at 95°C; 5 cycles of 30 s at 94°C, 30 s at 60°C and 90 s at 72°C; 25 cycles of 30 s at 94°C, 30 s at 56°C and 90 s at 72°C; and 7 min at 72°C; and for exons 7–9, 2 min at 95°C, 5 cycles of 30 s at 94°C, 30 s at 66°C and 90 s at 72°C; 25 cycles of 30 s at 94°C, 30 s at 62°C and 90 s at 72°C; and 7 min at 72°C. Genotyping was submitted on a regular basis to the external proficiency tests organized by the UCLA Immunogenetics Center (International KIR DNA exchange) to ensure its sensitivity, specificity and consistency, by means of comparison with the results obtained by other labs on samples distributed by the provider.

### Haplotype Assignment

**Figure 2:**
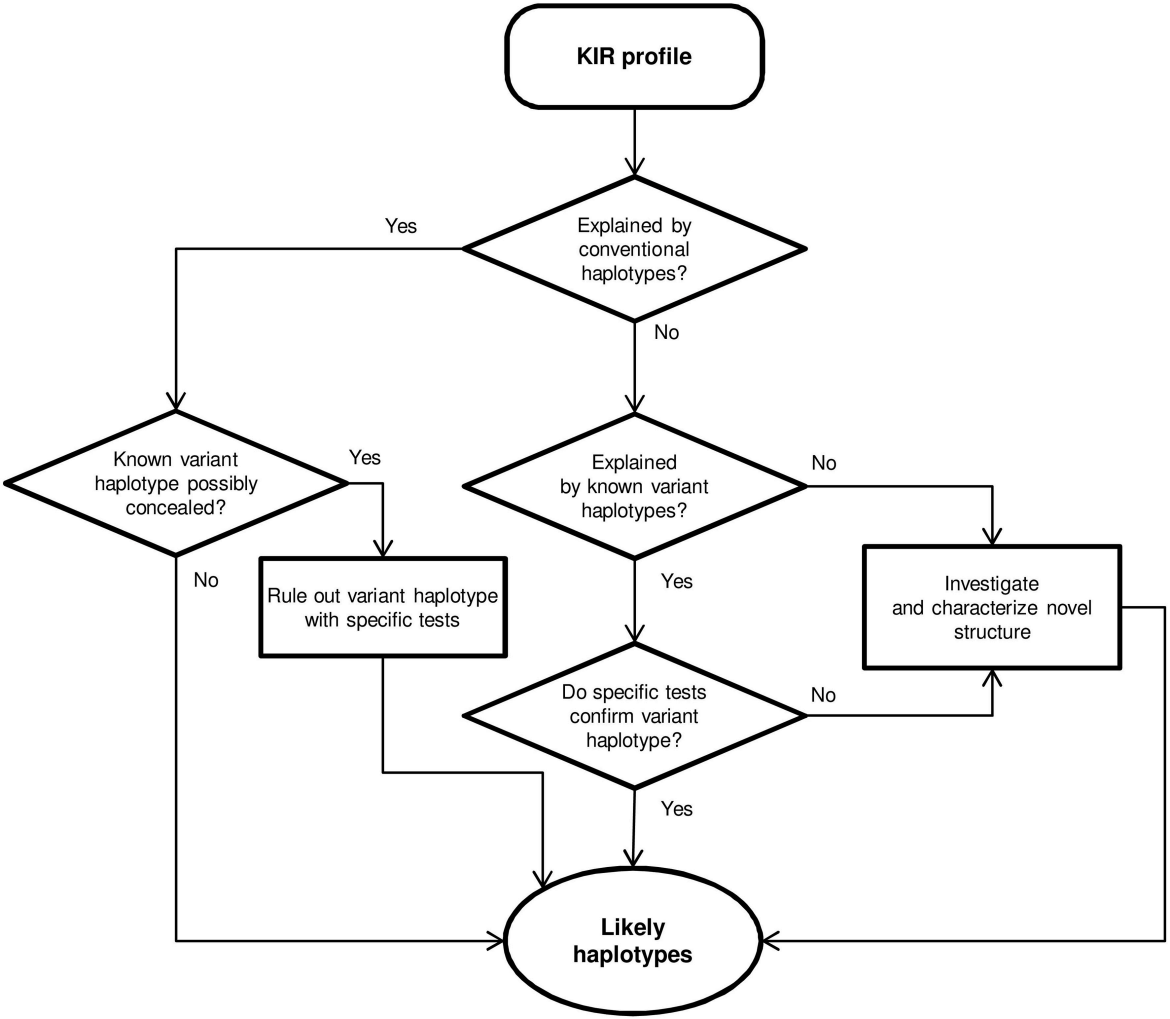
Flowchart for haplotype estimation from *KIR*-gene profiles.

Centromeric and telomeric *KIR*-gene arrangements were inferred in each individual by comparing their gene profile with the common and well-characterized haplotypes shown in Figure 1, assuming as few atypical or unknown combinations as possible. In particular, with the exceptions mentioned in the Results section, the following general assumptions were made, based on previous physical mapping and family segregation analyses, and on linkage disequilibrium (LD) between genes, confirmed in our samples using PHASE v2.1 software (46, 47) (results not shown): (i) *KIR 2DP1*-*2DL1*, and *3DL1*-*2DS4* are fixed blocks in complete linkage. (ii) Full and deleted *3DP1* variants mark cen-B2 and cen-A/cen-B1 haplotypes, respectively. (iii) Members of pairs *3DL1/3DS1*, and *2DL3/2DL2* behave as alleles of the same locus. (iv) Similarly, *2DS3* and *2DS5* were considered allotypes of a duplicated locus, associating invariably with *2DL5*. (v) The duplicated *2DL5-2DS3/S5* cluster was assigned to the centromeric or the telomeric sides, or both, according to presence or absence of adjacent genes: *2DS2-2DL2* and *2DP1-2DL1* (centromeric); and *3DS1* and *2DS1* (telomeric). (vi) Ambiguities derived from the latter rule were solved by *2DL5* subtyping and taking into account the fixed associations of 2DL5A^*^001 with *2DS5* and 2DL5A^*^005 with *2DS3* in tel-B1 and tel-B2 haplotypes, respectively (15, 41); besides those of *2DL5B* with 3DP1^*^003 and centromeric forms of *2DS3* (or, rarely in Caucasoids, *2DS5*). As shown in Figure 2, and detailed in Results, *KIR* gene profiles not fitting with these rules were then compared with contracted and expanded haplotypes described in detail by us and others, and presence of these or new arrangements was verified, when appropriate, by genotyping characteristic traits, such as hybrid genes, or characterized by *de novo* sequencing. Complete haplotypes were assigned only when linkage in cis of centromeric and telomeric motifs was unambiguous; a common ambiguity was presence of two different motifs on both the centromeric and the telomeric intervals, circumstance in which no complete haplotypes were assigned. Relative linkage disequilibrium (D′) between common centromeric and telomeric haplotypes, and its statistical significance were estimated with CubeX (48).

### Characterization of KIR2DL3^*^033

A new *KIR2DS2/2DL3* hybrid was identified by sequencing a partial genomic fragment amplified with primers for exons 5 and 9 (details available upon request). To fully characterize the new hybrid KIR, 2DL3^*^033, we amplified its complete gene by long-range PCR, using Advantage-2 polymerase mix; forward primer LFc−444 (5′–gctattctgatgcctctggtttagtac−3′), which recognizes a sequence conserved 5′ of most *KIR*, but not in previously known *2DL3* alleles; and the reverse primer LRt1375 (5′–caggagacaactttggatca−3′), specific for a stop codon unique of *2DL3*. PCR conditions were: 2 min at 95°C; 5 cycles of 20 s at 94°C, 30 s at 68°C and 15 min at 72°C; 30 cycles of 20 s at 94°C, 30 s at 64°C; and 15 min at 72°C. The ~14-Kbp amplicon, spanning from the 5′UT region to the stop codon, was sequenced with internal primers. Confirmatory sequences for the KIR2DL3^*^033 stop codon and its new polymorphisms in introns 6 and 7 were obtained from an additional 3.7-Kbp amplicon generated with forward primer Fi6t+1516, (5′–catcctaaagtactgggataact−3′, intron 6) and reverse primer Rt1460 (5′–acattggagctggcaaccca−3′, 3′UT), using the following PCR profile: 2 min at 95°C; 10 cycles of 20 s at 94°C, 30 s at 65°C and 4 min at 72°C; 20 cycles of 20 s at 94°C, 30 s at 61°C; and 4 min at 72°C.

To map KIR2DL3^*^033 within the KIR complex, gene walking (26, 41) was carried out – a ~7-Kbp amplicon spanning exons 7–9 of the preceding gene, the intergenic region and exons 1–4 of the target gene was generated by PCR for 2 min at 95°C; 30 cycles of 20 s at 94°C and 15 min at 72°C; and 20 min at 72°C with a *KIR*-generic forward primer (Fi6–81, 5′–ctaaagagacgttgtatgtggttacc−3′, intron 6) and the gene-specific reverse primer LRa546 (5′–ctccaatgaggtgcaaagtgtccttat−3′, exon 4).

Presence of KIR2DL3^*^033 in genomic DNA samples was screened by PCR-SSP, using BioTaq DNA polymerase (Bioline, London, UK) and primer mix Fi6t+1516/Ri6t+2713 (5′–catcctaaagtactgggataact−3′ and 5′–tctgtgctggaggattctga−3′), which recognizes a combination of polymorphisms unique to intron 6 of the *KIR2DS2/2DL3* hybrid, generating a 1387-bp amplicon. A primer pair recognizing a non-polymorphic sequence of the *COCH* gene served as an internal positive control of ~2 Kbp (COCH-Fi8–86, gaaagaaacttgtgtgttgtctggt; COCH-Ri11+95, attgggtaaagccacaggtgtttg). PCR conditions were: initial denaturation for 2 min at 95°C; 10 cycles of 20 s at 94°C, 30 s at 65°C and 90 s at 72°C; 20 cycles of 20 s at 94°C, 30 s at 61°C and 90 s at 72°C; and 7 min at 72°C.

### Genomic Characterization of KIR2DL5B^*^0020202

The complete coding region and part of the intervening introns of the new allele KIR2DL5B^*^0020202 were derived from a ~9.4-Kbp fragment generated from donor D139 by long range PCR with primer mix Fg−97b/Rg1769b (5′–tcaccctcccrtgatgtg−3′, promoter region; and 5′–ggaaggtggaacagcacgtgtctc−3′, 3′UTR) and Advantage-2 polymerase. PCR conditions were: 2 min at 95°C; 30 cycles of 20 s at 94°C and 15 min at 72°C; and 20 min at 72°C. The relative position of this allele in the *KIR* complex was determined by gene walking (26, 41). Identical procedures were used in another donor to identify and map KIR2DL5B^*^0020106 (32).

### DNA Sequencing and Nomenclature

PCR products were submitted, with no cloning step, to direct nucleotide sequencing in both strands, using internal primers (sequences available upon request). The products were analyzed in an ABI Prism 3100-Avant Genetic analyzer (Applied Biosystems) in the central DNA sequencing facility of *Instituto de Investigación Sanitaria Puerta de Hierro Segovia de Arana (IDIPHISA)*. The names KIR2DL3^*^033 and KIR2DL5B^*^0020202 (EMBL/GenBank/DDBJ database accession numbers HG931348 and LT604077, respectively) were officially assigned by the WHO Nomenclature Committee for factors of the HLA System, Subcommittee for Killer-cell Immunoglobulin-like Receptors (13).

### Flow Cytometry

The NK-cell population was defined in PBMCs by the CD3^−^CD56^+^ phenotype, using anti-CD3-VioBlue (Miltenyi Biotec, Bergisch Gladbach, Germany) and anti-CD56-APC (eBioscience, Inc, San Diego, CA). These were combined in a donor carrying KIR2DL3^*^033, with anti-KIR2DL3-FITC (180701, R&D Systems, Minneapolis, MN, USA) and anti-KIR2DL3/L2/S2-PE/Cy7 (DX27, Miltenyi Biotec); and in donors bearing transcribed KIR2DL5B^*^002 alleles, with anti-KIR2DL5-PE [UP-R1 (49), Biolegend, San Diego, CA, USA]. Isotype-matched negative controls were IgG1-PE (clone MOPC-21, Sigma-Aldrich, St. Louis, MO), IgG2a-FITC (clone MG2A01, Invitrogen, Camarillo, CA, USA), and IgG2a-PE (clone S43.10, Miltenyi Biotec). Flow cytometry analyses were performed in a MACSQuant Analyzer using MACSQuantify software (both by Miltenyi Biotec) in the central facility of *IDIPHISA*.

## Results

### *KIR*-Gene Frequencies and Profiles

To characterize and understand the diversity of *KIR* genotypes in the Spanish population, we determined the *KIR*-gene content in the genome of 414 unrelated donors using a locally designed PCR-SSP (sequence-specific primers) method, which also discriminates between structural variants of *KIR 2DS4* and *3DP1*. Unusual genotypes were further investigated and confirmed using a combination of techniques, including, as appropriate, probe hybridization; selective sequencing of the relevant coding, non-coding or intergenic regions; and physical mapping of neighbor genes (*KIR*-gene “walking”), when relevant.

Individual *KIR*-gene frequencies are shown in Table 1. Framework *KIR* genes and pseudogenes *3DL3, 3DP1, 2DL4*, and *3DL2* were detected in every donor, even though seven of them were then deduced to lack *3DP1-2DL4-3DL1/S1*, or have *3DL2* partially deleted, on one chromosome. Also found in all 414 donors was *KIR3DX1*, a gene of uncertain function located outside and 180 Kbp centromeric to the *KIR* complex, which is seldom typed for. Of note, only the latter gene and *KIR3DL3*, both of unknown biological significance (5, 50), appear to be truly conserved (i.e., not submitted to CNV) among human *KIR*.

**Table 1:** Carrier frequencies of *KIR* genes, pseudogenes, and their main structural and positional variants in 414 Spanish donors.

**Long tailed**			**Short tailed**			**Others**		
**Gene**	**Variant**	**%**	**Gene**	**Variant**	**%**	**Gene**	**Variant**	**%**
*2DL1*		96.6	*2DS1*		42.8	*2DP1*		96.6
*2DL2*		58.0	*2DS2*		58.7	*3DP1*	*All*	100.0
*2DL3*		88.4	*2DS3*	*All*	34.8		*Exon 2^+^*	31.4
*2DL4*		100.0		*Centromeric*	31.2		*Exon 2^*del*^*	96.6
*2DL5*	*All*	57.7		*Telomeric*	15.2	*3DX1*		100.0
	*Centromeric*	32.1	*2DS4*	*All*	95.9			
	*Telomeric*	41.5		*Correct CDS*	36.2			
*3DL1*		96.1		*Frame-shifted*	82.1			
*3DL2*		100.0	*2DS5*	*All*	30.0			
*3DL3*		100.0		*Centromeric*	0.0			
				*Telomeric*	30.0			
			*3DS1*		42.5			

*CDS, coding sequence*.

Non-framework genes encoding long-tailed KIR typical of A-haplotypes *2DL1, 2DL3*, and *3DL1* had frequencies of ~90%; whilst *2DL2* and *2DL5*, encoding inhibitory KIR characteristic of B-haplotypes were seen in ca. 60% of the donors. Activating *KIR*-gene frequencies were more variable, ranging from 30.0% (*2DS5*) to 95.9% (*2DS4*). The latter was most often represented by frame-shifted alleles (82.1 vs. 36.2% alleles with canonical coding sequence), therefore the most common functional activating *KIR* was actually *2DS2* (58.7%) (Table 1).

**Figure 3:**
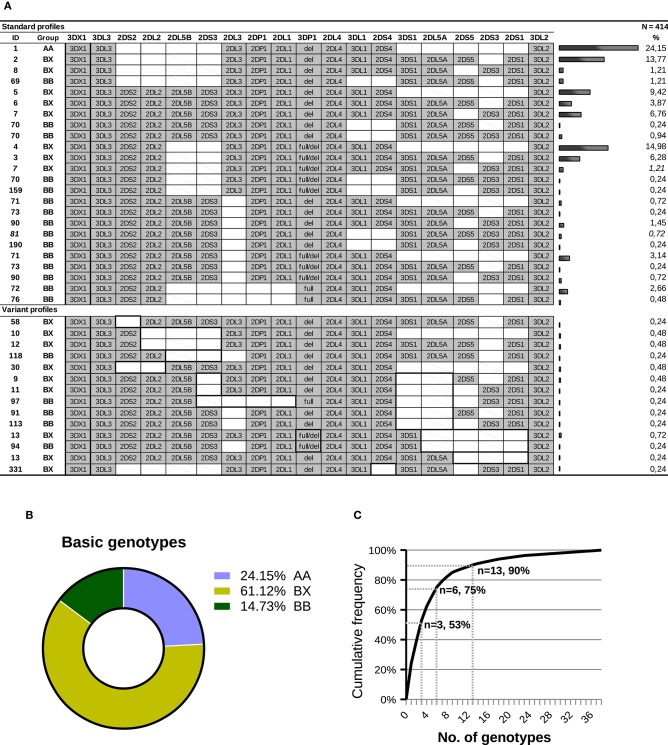
*KIR-*gene profiles observed in a sample of Spanish individuals. **(A)** Gene presence or absence is represented by solid gray and empty boxes; allelic forms are indicated for *KIR3DP1*. Carrier frequency is given on the right side of each genotype. Gene order reflects, approximately, that seen in the *KIR* complex, with genes forming A-haplotypes in the middle, flanked on both sides by genes characteristic of B-haplotypes. For *KIR 2DL5* and *2DS3*, genes represented by two paralogues, a diagonal line indicates that the gene is present in a genotype, but most likely in the alternative location. The ID column shows the number by which the genotype is registered in www.allelefrequencies.net (40); since this database does not distinguish between structural/positional variants, a same ID can correspond to several profiles in the table. Two profiles (7 and 81) include each one individual bearing a concealed variant haplotype, as explained in the text. In the lower part of the panel, which compiles gene profiles not explained by conventional haplotypes, thick lines highlight distinctive traits, including missing genes normally associated with ones present in a given genotype. **(B)** Distribution of the major groups of *KIR* genotypes. **(C)** Cumulative frequencies of *KIR* profiles.

Based on *KIR*-gene content, we found 38 different profiles, which were sorted into three groups: (i) the AA profile carrying exclusively genes of the A haplotype (24.15% of individuals); (ii) 16 BX profiles having all genes of the A-haplotype plus one or more B-haplotype genes (61.12%); and (iii), 21 BB profiles, defined by presence of B-haplotype genes and lack of one or more genes of the A-haplotype (14.73% of individuals). The individual and grouped frequencies of those profiles are shown in Figures 3A,B. The AA genotype was most frequent (ID: 1, 24.15%), followed by six BX genotypes (ID: 4, 2, 5, 7, 3 and 6) which, together, account for more than 50% of individuals; and by the two most common BB profiles (ID: 71 and 72; 3.14 and 2.66%, respectively). The overall distribution of *KIR*-gene frequencies and profiles observed in our sample is not dissimilar from those reported in other Caucasoid populations, and it is also consistent with those found in other large samples of Spanish individuals (40, 51–53).

A cumulative frequency analysis of the distribution of *KIR* genotypes is shown in Figure 3C. More than 50% of the population can be represented by three genotypes; six genotypes account for 75% of the total; and 13 are needed to explain 90% of the diversity. The remaining 10% is accounted for by 25 gene profiles, 14 of which were observed only once. As analyzed in more detail below, more than one third of the gene profiles (fourteen genotypes, belonging to 20 individuals) cannot be explained by any homo- or heterozygous combination of canonical haplotypes.

### Centromeric and Telomeric Interval Analysis

**Figure 4:**
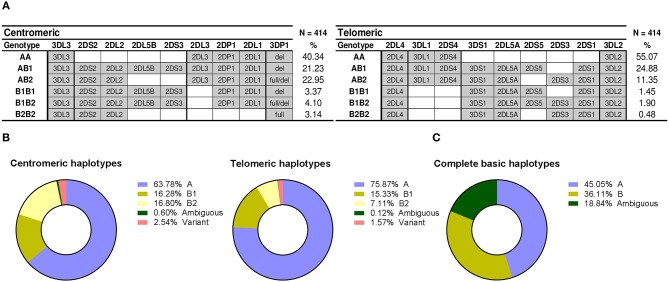
Distribution of centromeric and telomeric gene profiles and haplotypes. **(A)** Centromeric and telomeric profiles corresponding to combinations of conventional haplotypes are represented as in Figure 2. Profiles derived from variant and novel arrangements are not represent, therefore frequencies do not sum up 100%. **(B)** Distribution of partial *KIR* haplotypes. **(C)** Frequencies of complete haplotypes and phasing ambiguities.

Following a parsimonious approach that assumed as few non-canonical or novel *KIR*-gene arrangements as possible, we assigned each gene profile to the most likely diploid combination of well-known centromeric and telomeric haplotypes seen in most ethnicities (Figure 1). This was possible in 394 donors (95.17%), and the frequencies of their centromeric and telomeric deduced genotypes are represented in Figure 4A. On the centromeric region, the cen-AA genotype was most common (40.34%), followed by combinations of cen-A with either cen-B1 or cen-B2 haplotypes, seen with even frequencies-−21.23 and 22.95%, respectively. Much less frequent were combinations of only cen-B1 or -B2 motifs, each seen at <5%. Centromeric motifs consistently associated with the major *KIR3DP1* allotypes (exon 2^+/del^) as reported (54), with a single exception described previously by us [haplotype *2DS2-2DL2-3DP1*^*del*^ in family C180 (43)]. On the telomeric side, the combination of two tel-A segments was again most common at 55.07%; followed by combinations of tel-A and tel-B motifs (24.88% tel-AB1 and 11.35% tel-AB2); whereas combinations of tel-B1 or -B2 haplotypes collectively represented only 3.83% of telomeric genotypes.

From the previous genotypes, and from those eventually assigned to individuals with unconventional profiles (following section), we estimated the frequencies of individual centromeric and telomeric haplotypes (Figure 4B). Also based on those assignments of partial centromeric and telomeric motifs, entire basic haplotypes (i.e., A vs. B) could be phased in 81% of donors (i.e., those who, on at least one of their telomeric or centromeric segments, only had either A or B profiles, but not both). This allowed a minimum estimate of the frequency of such complete basic haplotypes (Figure 4C): at least 45% of haplotypes are A (cen-A/tel-A), whilst more than 36% of haplotypes would be of the B-type (cen-A/tel-B, cen-B/tel-A, or cen-B/tel-B). The remaining ~19% of haplotypes could not be unambiguously assigned to the A- or the B-groups, because coincidence of A and B profiles on both the centromeric and the telomeric regions of the same individual precluded phasing.

Linkage disequilibrium between common centromeric and telomeric motifs is analyzed in Table 2, and depicted in Figure 1. Of note, association between cen-A and tel-A haplotypes was weak (D' = 0.089) and non-significant, suggesting that their common composition of a complete A haplotype is explained merely by the high frequency of its two segments. In contrast, strong were the positive LD of tel-B2 (*KIR 3DS1-2DL5-2DS3-2DS1*) with cen-B1, and its negative LD with cen-A (0.70 and −0.73, respectively; *p* < 0.0001). As previously noted (17), this means that the telomeric cluster 2DL5A^*^005-2DS3^*^002 is most often associated with a nearly identical sequence (2DL5B^*^002-2DS3^*^001) on the centromeric segment of the same haplotype, perhaps a reminiscence of their recent common origin.

**Table 2:** Linkage disequilibrium between centromeric and telomeric *KIR* haplotypes.

**hap. freq. D^**′**^ p**	**tel-A**	**tel-B1**	**tel-B2**
cen-A	0.503	0.129	0.012
	0.089	**0.546**	**−0.727**
	n.s.	<0.05	<0.0001
cen-B1	0.096	0.012	0.054
	**−0.253**	−0.534	**0.702**
	<0.01	n.s.	<0.0001
cen-B2	0.155	0.011	0.003
	**0.692**	−0.574	−0.775
	<0.05	n.s.	n.s.

*N.s., non-significant. Bold, statistically significant D' values*.

Taken together, only 24 of the 38 observed *KIR*-gene profiles were explained by combinations of canonical centromeric and telomeric motifs; whilst no such combination could account for 14 profiles (i.e., more than one third), which owed to 2.66% of all chromosomes carrying atypical *KIR*-gene arrangements, as analyzed in the following sections.

### *KIR* Gene Profiles Explained by Known Non-canonical Arrangements

**Figure 5:**
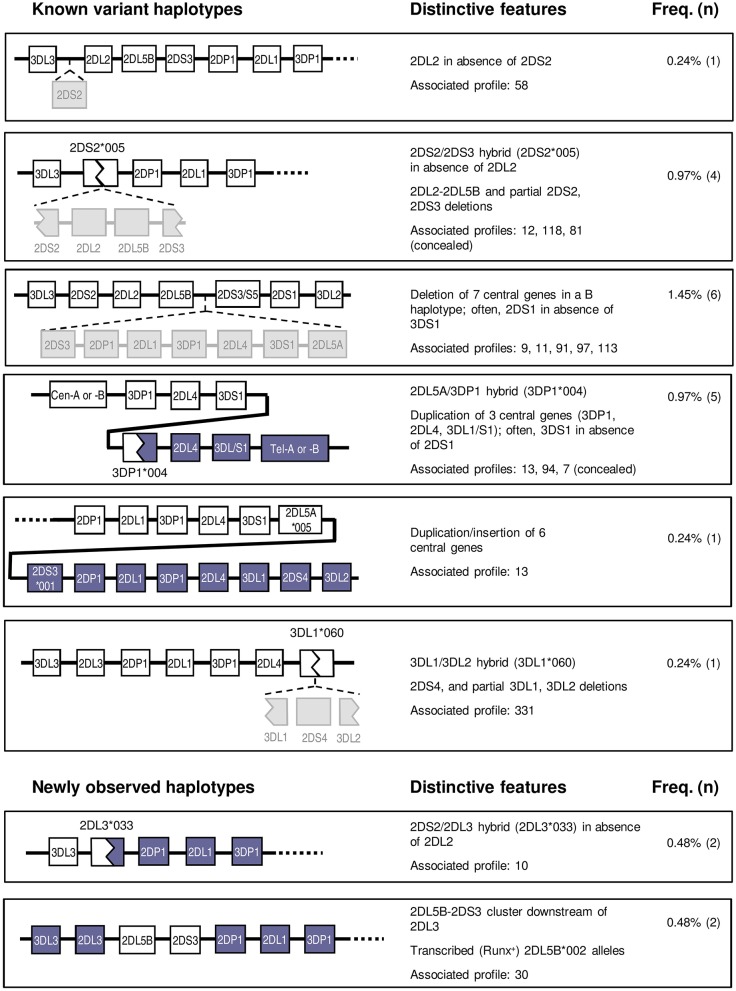
Variant and novel haplotypes detected in Spanish individuals. Colors are used to highlight gene deletions and hybrid structures derived from recombination of two different genes or haplotypes, but they lack a specific meaning. Duplicated genes are represented in parallel.

Twenty individuals (4.8%) had *KIR* genotypes unexplained by any homo- or heterozygous combination of conventional haplotypes. Of those, 17 could be explained, as detailed below, by recombinant or variant structures previously described by us and others. Furthermore, specific tests targeting marker polymorphisms disclosed two additional donors in whom atypical haplotypes were concealed under apparently canonical *KIR* profiles, making a total of 22 individuals (5.3%) with unusual *KIR*-gene arrangements (Figure 5). Finally, three donors carried new haplotypes or hybrid genes characterized in the following sections.

#### Centromeric Interval

Two atypical profiles with singularities affecting only the centromeric region were found in seven donors. One of them presented *KIR2DL2* in absence of *2DS2*. This profile, initially characterized in Black individuals (8, 19), might be explained by *KIR2DS2* deletion from the usual *2DS2-2DL2* cluster; alternatively, since these two highly homologous genes possibly derive from a common ancestor, the variant profile might be reminiscent of the ancestral haplotype existing before the gene duplication that gave origin to the two paralogues (Figure 5).

Another five individuals had the reciprocal combination, i.e., *KIR2DS2* in isolation from *2DL2* and/or other cen-B1 characteristic genes, a profile typically seen in a shortened haplotype previously characterized by our group (43). Such haplotype, generated by unequal crossing-over between *KIR2DS2* and *2DS3*, is marked by the resulting chimeric allele, KIR2DS2^*^005, and by deletion of the intervening genes (*KIR2DL2, 2DL5B*, and *2DS3)*. A PCR-SSP test specific for KIR2DS2^*^005 confirmed this marker in three of the five suspected individuals; and disclosed it in another donor with an apparently standard *KIR* profile, in which lack of *KIR2DL2* within the recombinant haplotype was concealed by its presence on the other one.

The remaining two *KIR2DS2*^+*ve*^*-2DL2*^−*ve*^ individuals did not carry this shortened B haplotype, since they were negative in the KIR2DS2^*^005 test. Instead, they carried a new recombinant haplotype and a novel hybrid gene, as analyzed in a separate section. Also described separately is the last variant centromeric profile, in which the *KIR 2DL5B-2DS3* cluster is seen in absence of its usual companions *2DS2-2DL2*.

#### Central Cluster

Affecting both the centromeric and the telomeric regions were haplotype arrangements that included duplications or deletions of the central framework genes, identified in 13 donors. For instance, an atypical profile marked by presence of *KIR 2DS3/2DS5* and *2DS1* in absence of *3DS1* was explained by previously characterized haplotypes bearing an extensive deletion affecting seven genes on the central region of the *KIR-*gene complex, including, among others, the otherwise conserved *KIR 3DP1, 2DL4*, and *3DL1/3DS1* loci (19, 20). The resulting contracted B haplotype, containing only seven genes, generates an unusual juxtaposition of the centromeric *KIR2DL5B* and the telomeric *KIR2DS3/2DS5* loci. This profile was observed in six individuals, but in contrast with studies on other ethnic groups, the deletion of the central genes was never observed in homozygosis in this population sample. However, such genotype does exist among Spanish Caucasoids, as we reported earlier [donor LH304 (49)].

Five individuals showed the opposite combination—*KIR3DS1* in absence of *2DS1* (and the telomeric *2DL5-2DS5/S3* group). This trait is typically seen associated with a tandem duplication of the *3DP1-2DL4-3DL1/S1* cluster and the recombinant, transcribed KIR3DP1^*^004 allotype, a marker of this duplication (21, 22). This “full” (exon 2^+*ve*^) *3DP1* allotype is often discordant at first glance with the centromeric profile, serving as a beacon of the expanded haplotype. Its presence in four of the five suspected individuals, as well as in one with an apparently normal genotype, was confirmed by specific PCR-SSP detection of the hybrid gene KIR3DP1^*^004.

A sixth donor with a similar *KIR* genotype (*3DS1*^+*ve*^*-2DL5*^+*ve*^*-2DS3*^+*ve*^*-2DS1*^−*ve*^) was, in contrast, negative for 3DP1^*^004. Its atypical gene profile could instead be explained by presence of an expanded haplotype we described in one Irish Caucasoid (17). Such haplotype is characterized by an even larger duplication/insertion of six central *KIR* genes, due to unequal crossing-over between the intergenic regions of the telomeric and the centromeric *2DL5-2DS3* clusters (Figure 5). Consistently with such duplication, the donor had two *3DL1* alleles besides *3DS1* (not shown).

#### Telomeric Interval

Least common were variants affecting exclusively the telomeric *KIR* region—we observed a single unusual *3DL1*^+*ve*^-*2DS4*^−*ve*^ profile, represented in one donor. In this individual, we identified another previously described hybrid gene, KIR3DL1^*^060, arising from an asymmetric recombination event that fused *3DL1* exons 1–5 with *3DL2* exons 6–9 (44, 45). Concomitant deletion of the intervening gene *KIR2DS4* explains this gene profile.

### A Novel *KIR2DS2/KIR2DL3* Fusion Gene Within a Recombinant B/A Motif Challenges Conventional Gene and Haplotype Classification

One donor apparently presented *KIR2DS2* in isolation from other B-haplotype genes (i.e., in the context of an AA genotype), but, as stated earlier, was negative for 2DS2^*^005, which marks the only known gene arrangement that could account for such profile (43). To characterize the putative *2DS2* gene in the novel gene profile, we amplified its exons 5 through 7 (encoding the D2 Ig-like domain, stem and transmembrane region) in a 7.6-Kbp genomic fragment. Analysis of this amplicon revealed, instead of a proper *KIR2DS2* gene, a new hybrid sequence, of which the 5′- and the 3′-ends were homologous to *2DS2* and *2DL3*, respectively. Based on this information, the new gene was then amplified in a single ~14-Kbp genomic fragment comprising all its exons and introns, and sequence analysis confirmed its hybrid nature—its 5′ side (down to nucleotide 2,075 of intron 6, ca. 11 Kbp) was identical to 2DS2^*^0010102; whilst the rest of the gene (~3 Kbp through the stop codon) matched 2DL3^*^0020101, except for three base substitutions: two unique in intron 6 (11586 A>G and 11849 C>T), and one in intron 7, shared with 2DL3^*^010 (13627 G>A). The hybrid sequence is likely the result of asymmetric (non-allelic) homologous recombination between *KIR2DS2* and *KIR2DL3* genes present in different B and A haplotypes, respectively. The apparent recombination spot is few bases upstream of an AluSX element (not shown).

As discussed later, the novel structure challenged unwritten rules followed previously to designate chimeric KIR, not sitting comfortably within any of the current designations. It was officially assigned to the *KIR2DL3* locus with the name 2DL3^*^033 by the KIR Nomenclature Committee, which reflects appropriately the fact that the encoded protein should have an intracellular inhibitory tail identical to those of most other *2DL3* alleles.

To map the new hybrid gene, we used a *KIR*-gene walking approach (i.e., amplification and sequencing of a genomic fragment spanning part of the gene of interest, and one in its vicinity), which showed 2DL3^*^033 to be located 3′ of *3DL3*, like common alleles of both its homologs *2DS2* and *2DL3* in conventional *KIR* haplotypes. Furthermore, the donor had 3DL3^*^003, allele commonly linked to *2DS2* (24), in consonance with presence of a *2DS2*-like 5′ region in 2DL3^*^033.

**Figure 6:**
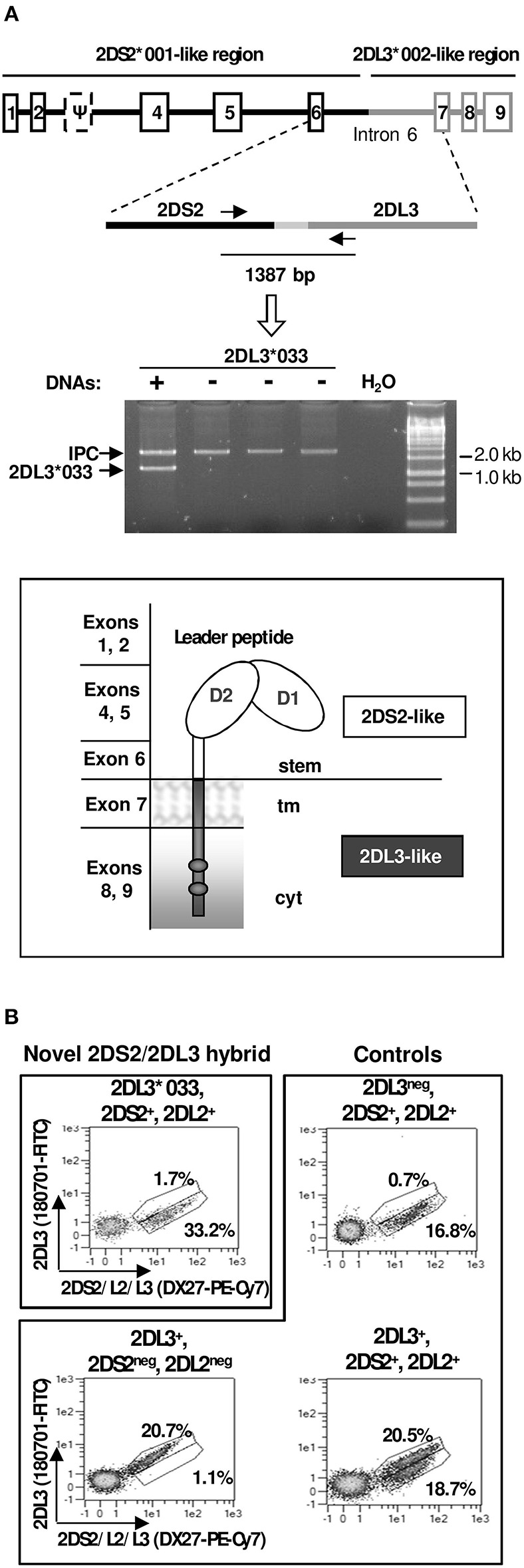
Characterization of KIR2DL3*033. **(A)** Gene and protein structure, including homology to other KIR, and an example of the PCR-SSP test to identify the novel allele (lane 1). IPC stands for internal positive control. **(B)** Flow cytometry plots of NK cells (CD3^−^CD56^+^) from a donor expressing KIR2DL3*033, and from others with common *KIR 2DL2/2DS2/2DL3* genotypes.

PBMCs of the donors in whom KIR2DL3^*^033 was originally found were, unfortunately, unavailable. To estimate the KIR2DL3^*^033 distribution and enable expression studies on this allele, we designed a PCR-SSP method targeting a specific combination of polymorphisms in its sixth intron, which led us to identify two additional examples of this allele in 1,101 DNA samples (~0.2%; confidence interval with *p* < 0.05: 0.00–0.46%). Using PBMC of one of those donors, we could readily amplify the KIR2DL3^*^033 complete coding region (~1.1 kb) by RT-PCR, demonstrating the normal transcription and processing of its mRNA. According to this, KIR2DL3^*^033 encodes ligand-recognition Ig-like domains, and a stem homologous to those of the activating 2DS2, but transmembrane and long intracytoplasmic regions like those of 2DL3 (Figure 6A). Therefore, the encoded receptor should combine the weak HLA-C1 recognition of the former, and the inhibitory capacity of the latter.

Since PBMCs of KIR2DL3^*^033 donors with cenAA profiles were unavailable, we performed multicolour flow cytometry analyses of PBMCs of one available individual encoding KIR2DL3^*^033 on one haplotype and the 2DS2-2DL2 combination on the second one. The observed lack of staining with a KIR2DL3-specific mAb (Figure 6B) was consistent with the 2DS2-like nature of the KIR2DL3^*^033 ectodomain. Unfortunately, the donor genotype precluded positive identification of cells expressing KIR2DL3^*^033, since these are indistinguishable with the available mAbs from those bearing its homologues 2DS2 and 2DL2. Flow cytometry studies of further donors with favorable (i.e., cenAA) *KIR* profiles are warranted to demonstrate positively KIR2DL3^*^033 NK-cell surface expression; this would be marked by presence of cells staining with DX27 (or equivalent mAbs), but not with reagents monospecific for conventional KIR2DL3 alleles.

### KIR2DL5B^*^0020202—A Transcribed Allele Linked to *KIR2DL3* in an Unusual Centromeric B Motif

Two individuals exhibited unusual presence of the *KIR 2DL5-2DS3* genes in absence of any other genes characteristic of *B* haplotypes, to which they are normally linked (i.e., *2DL2* or *3DS1*). To understand the unusual genotype, we used *KIR*-gene walking—amplification of a ~4-Kbp region spanning *KIR2DL5* exons 1–3, the last (ninth) exon of the unknown preceding gene, and the intergenic region. This enabled us to map, in both donors, KIR2DL5B^*^002-related sequences downstream of KIR2DL3^*^010. This arrangement is seemingly identical to one previously described in a minority of Black Africans (8, 9, 32, 55), in whom a *2DL5-2DS3* block is inserted between *2DL3* and *2DP1-2DL1* (Figure 5), thus converting a centromeric A-motif into a B-haplotype, according to the agreed definition of these.

**Figure 7:**
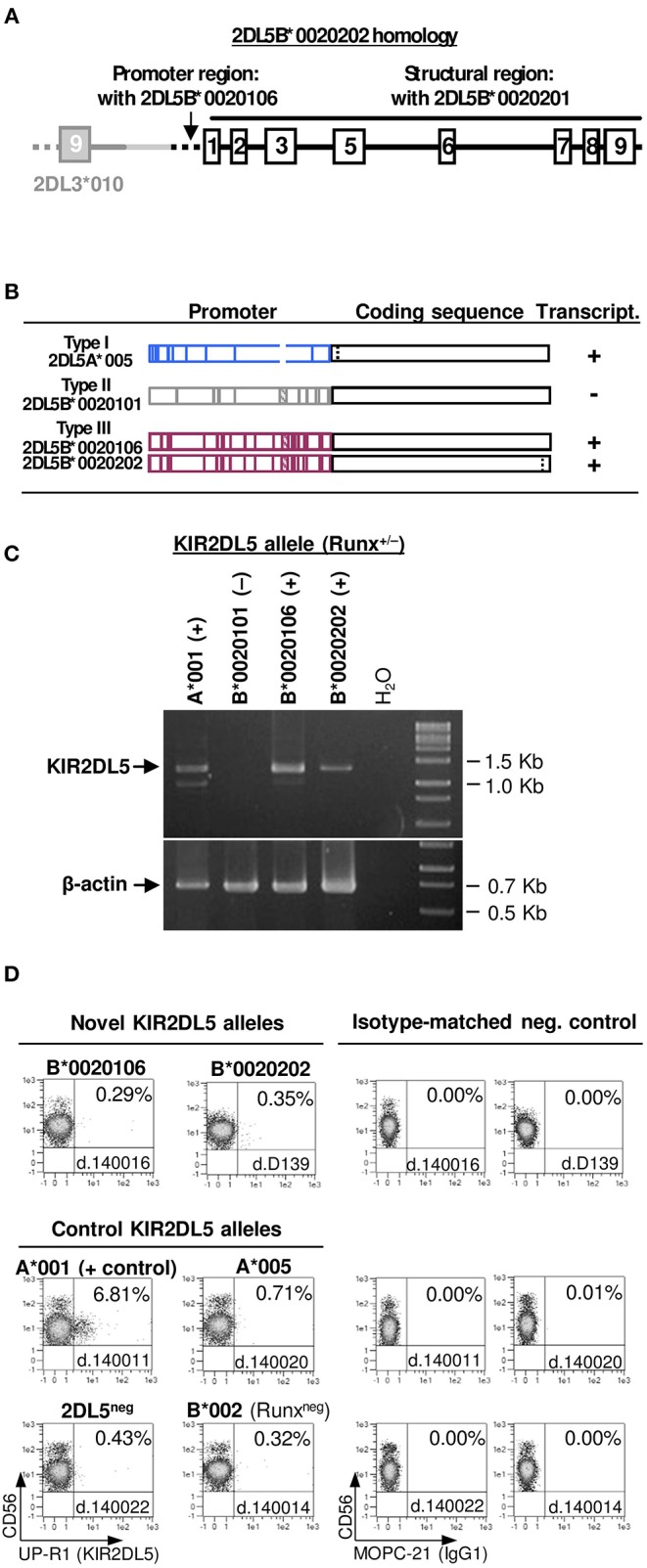
Characterization of KIR2DL5B*0020106 and *0020202. **(A)** Gene structure of KIR2DL5B*0020202, including homology to other *KIR2DL5* alleles. **(B)** Comparison of *KIR2DL5* alleles carrying nearly identical coding sequences but highly divergent promoter regions; vertical lines represent polymorphisms distinguishing those alleles. Gene transcription or silencing is indicated on the right side. **(C)** RT-PCR assay showing KIR2DL5B*0020106 and *0020202 transcription, in contrast with their common, silent homologue KIR2DL5B*0020101. Presence or absence of an intact RUNX binding site in the proximal promoter is indicated for each allele. **(D)** A KIR2DL5 product is undetectable on the surface of NK cells transcribing KIR2DL5B*0020106 and *0020202, as we reported previously for KIR2DL5A*005, which encodes an identical mature polypeptide.

To further characterize *KIR2DL5B* in the unusual haplotype, we amplified the whole gene by long-range PCR (~9 Kbp). Sequence analysis of this amplicon, along with that derived from *KIR*-gene walking, revealed, in one donor, a new *KIR2DL5B* allele, designated KIR2DL5B^*^0020202, in which a coding region identical to that of the previously known KIR2DL5B^*^0020201 is fused to a “*KIR2DL5*-type III” promoter (15), similar to that found in the expressed allele KIR2DL5B^*^003 (Figure 7A). This type of promoter retains an intact RUNX-binding site seen in all clonally expressed *KIR*, in contrast with common *KIR2DL5B* alleles, in which the site is destroyed by substitution of adenosine for guanosine −97, polymorphism completely associated with epigenetic silencing (41). Similar results were obtained in the second donor, in whom we found allele KIR2DL5B^*^0020106. This allele and the novel KIR2DL5B^*^0020202 share a nearly identical combination of promoter and coding sequences, the latter differing by a single synonymous substitution (Figure 7B).

Conservation of the RUNX site in KIR2DL5B^*^0020106 and ^*^0020202 should, according to our hypothesis (41), confer these alleles a capacity to be transcribed, in contrast with most *2DL5B* alleles. To test this prediction, we performed RT-PCR experiments on RNA isolated from PBMC of donors D139 and 140016. Specific, correctly spliced amplicons were readily obtained for both KIR2DL5B^*^0020106 and KIR2DL5B^*^0020202 (Figure 7B), as verified by direct sequencing. This positive result opened the possibility of KIR2DL5B being expressed on the cell surface, which has never been shown. To explore this, we undertook flow-cytometry assays with the KIR2DL5-specific mAb UP-R1, but these showed no specific surface staining of peripheral blood NK cells (Figure 7C). This result is in line with the described behavior of allele KIR2DL5A^*^005, which shares with KIR2DL5B^*^002 the identical sequence in the mature protein. Such protein, seemingly due to substitution of Ser for Gly174, is retained intracellularly in NK cells, besides reacting weakly with mAb UP-R1 (56); unfortunately, monoclonal antibodies for intracellular KIR2DL5A^*^005/B^*^002 staining are unavailable.

## Discussion

In-depth genomic studies have established the variable organization of the human *KIR* complex, defining gene motifs and extended haplotypes fixed in our species, and a series of variations from those, mostly generated by asymmetric (non-allelic) homologous recombination (4, 6–10, 26, 28, 29). In addition, sequence analyses based on Sanger and, more recently, second generation methods, have revealed the diversity of alleles commonly found in each of those *KIR* haplotypes (23, 24, 30–32, 34–39).

In parallel, the *KIR*-gene profiles of multiple populations worldwide have been explored in the last two decades, revealing that, whilst many *KIR* haplotypes and alleles are shared by humans of all ethnicities, notorious differences in their distribution exist, unveiling the evolutionary connections between human groups, and the variable selective pressures exerted on them by the environment (16, 40). However, it is noteworthy that many published population studies have not benefited sufficiently from knowledge on *KIR* polymorphism gained from genomic studies, often reporting only rough analyses of gene content and basic classifications on the “B-ness” or “A-ness” of *KIR* haplotypes.

We have tried to contribute to fill that gap by studying a sample of Spanish individuals by means of: (i) A combination of rapid and advanced methods for *KIR*-gene profiling that inform, not only of presence/absence of *KIR* genes, but also of isoforms associated with defined haplotypes, and recombinants that mark contracted/expanded haplotypes; (ii) Parsimonious interpretation of *KIR*-gene profiles based on accumulated knowledge on common and well-defined haplotypes and alleles; and (iii) Basic molecular characterization of a minority of *KIR*-gene profiles not fitting with known arrangements. Limitations of our study are that polymorphism has, in general, not been defined at an allelic level; and that our parsimonious approach might theoretically overlook part of the existing diversity. To mitigate the latter limitation we have screened certain specific recombinations in donors with compatible profiles, thus identifying individuals in whom variant structures were concealed by the accompanying genes.

By applying systematically this approach, we have managed to explain all 38 gene profiles found in 414 individuals, inferring their haplotype structures and incorporating them to analysis of *KIR*-gene distribution. We have thus determined the detailed distribution of the three fixed centromeric and three telomeric motifs; of six expanded or contracted *KIR*-gene arrangements characterized previously by us and others, seen in 22 individuals (5.31%); and two novel haplotypes never detected before in Caucasoids. These novel arrangements are associated with new *KIR* alleles, and they show combined features of B- and A-haplotypes.

Transcribed KIR2DL5B^*^002 alleles, contrasting with common silent ones, were found within a *2DL5B*-*2DS3* cluster inserted in a centromeric *2DL3*-*2DP1*-*2DL1* motif, thus converting it in a cen-B haplotype. Such structure had been previously reported in individuals of African origin (8, 9, 32). Transcription of KIR2DL5B^*^0020106 and ^*^0020202 provides further confirmation to our hypothesis that an intact RUNX binding site in the proximal promoter is essential for *KIR*-gene expression, whilst its mutation determines epigenetic silencing. The biological significance of transcribed KIR2DL5B^*^002 alleles is, however, uncertain since, like KIR2DL5A^*^005 (Figure 7), the encoded receptor appears not to reach the cell surface and is possibly retained intracellularly (56). The fact that nearly identical coding sequences are preceded by three highly divergent promoter sequences in alleles of two paralog genes (2DL5A^*^005, 2DL5B^*^0020101 and 2DL5B^*^0020106/0020202) illustrates the role of recombination in *KIR*-gene evolution.

The second novel allele, KIR2DL3^*^033, and its associated haplotype do not fit comfortably with the current classification and designation of *KIR* genes and haplotypes, posing a puzzling nomenclature issue. Its long inhibitory tail, homologous to that of common *KIR2DL3* alleles, should warrant its designation as a *KIR2DL*. Such name, however, challenges an unwritten rule of assigning hybrid *KIR* to the locus contributing the extracellular portion (e.g. 2DS2^*^005, a *2DS2/2DS3* hybrid, or 3DL1^*^060, a *3DL1/3DL2* chimera), and it obviates that most of the gene (~11 of 14 Kbp) and of the encoded molecule (all the ectodomain) are actually identical to those of *KIR2DS2*, and will be detected as such by most current genotyping and phenotyping assays. This may result in ambiguous or conflicting profiles, which can be sorted out by assays that, like the one we have used (Figure 6A), target specifically the KIR2DL3^*^033 recombination spot in intron 6. Furthermore, whereas recombinations of B- and A-haplotypes normally yield B-haplotypes, gene content of the hybrid haplotype bearing 2DL3^*^033 adjusts to the definition of A-haplotypes, its “B-ness” being perceived only when the actual sequence of its centromeric genes (*KIR 3DL3* and *2DL3*) is considered. On the other hand, assigning the new structure to the *KIR2DS2* gene would better reflect its origin, but would imply designating a long-tailed KIR with an “S” symbol aimed at distinguishing short-tailed, activating, KIR. To circumvent such and similar inconsistencies, the official KIR nomenclature might consider in the future the use of dedicated names to describe the hybrid nature of recombinant KIR genes (e.g., KIR2DS2/L3, KIR3DL1/L2, et cetera).

In summary, we consider that our study provides a representative and precise estimate of the KIR structures seen in the Spanish population, and extends our understanding of their complexity in South European Caucasoids. We expect that our results, and the approach we followed to obtain them, will enable better-founded studies of KIR in populations, and in health conditions in which their genetic diversity is deemed relevant.

## Data Availability Statement

The datasets generated for this study can be found in the EMBL/GenBank/DDBJ HG931348, LT604077.

## Ethics Statement

The studies involving human participants were reviewed and approved by Comité Ético de Investigación con Medicamentos, Hospital Universitario Puerta de Hierro, Majadahonda. The patients/participants provided their written informed consent to participate in this study.

## Author Contributions

EC designed and performed experiments, analyzed and interpreted data, and wrote the manuscript. MM performed experiments, analyzed and interpreted data, and revised the manuscript. NG-L, AM, and ML-B contributed samples, and revised the manuscript. CV designed the study, directed research and wrote the manuscript.

### Conflict of Interest

The authors declare that the research was conducted in the absence of any commercial or financial relationships that could be construed as a potential conflict of interest.
